# Supplementary material for: Calm before the Storm: A Glimpse into the Secondary Metabolism of Aspergillus welwitschiae, the Etiologic Agent of the Sisal Bole Rot
Source: Toxins (Basel). 2019 Oct 30;11(11):631. doi: 10.3390/toxins11110631 (PMC6891411; doi:10.3390/toxins11110631)
Supplement: Supplementary file 1 [file toxins-11-00631-s001.pdf]

Supplementary Material: Calm Before the Storm: A Glimpse into the Secondary Metabolism of *Aspergillus welwitschiae*, the Etiologic Agent of the Sisal Bole Rot

Gabriel Quintanilha-Peixoto, Rosimére Oliveira Torres, Isabella Mary Alves Reis, Thiago Alves Santos de Oliveira, Dener Eduardo Bortolini, Elizabeth Amélia Alves Duarte, Vasco Ariston de Carvalho Azevedo, Bertram Brenig, Eric Roberto Guimarães Rocha Aguiar, Ana Cristina Fermino Soares, Aristóteles Góes-Neto and Alexsandro Branco

Table S1. Fractions were obtained (Table S1) and analyzed in HPLC coupled to a photodiode array. Yield value refers to initial 0.87 g.

Fraction	Solvent System (%)	Mass (g)	Yield (%)
Fr-1	Hexane (100)	0,0139	1,61%
Fr-2	Hexane (100)	0,0273	3,15%
Fr-3	Hexane: Ethyl acetate (80:20)	0,0998	11,52%
Fr-4	Hexane: Ethyl acetate (70:30)	0,0374	4,32%
Fr-5	Hexane: Ethyl acetate (60:40)	0,0175	2,02%
Fr-6	Hexane: Ethyl acetate (50:50)	0,0155	1,79%
Fr-7	Hexane: Ethyl acetate (50:50)	0,0234	2,70%
Fr-8	Hexane: Ethyl acetate (50:50)	0,0134	1,55%
Fr9-10	Hexane: Ethyl acetate (40:60)	0,0101	1,17%
Fr-11	Hexane: Ethyl acetate (30:70)	0,0089	1,03%
Fr-12	Hexane: Ethyl acetate (10:90)	0,0133	1,54%
Fr-13	Ethyl acetate (100)	0,0170	1,96%
Fr-14	Ethyl acetate: Methanol (80:20)	0,0204	2,36%
Fr15-16	Ethyl acetate: Methanol (70:30)	0,0197	2,27%
Fr-17	Ethyl acetate: Methanol (50:50)	0,0123	1,42%
Fr18-19	Ethyl acetate: Methanol (30:70)	0,0489	5,65%
Fr-20	Ethyl acetate: Methanol (20:80)	0,0080	0,92%
Fr-21	Methanol (100)	0,0055	0,64%
Fr-22	Methanol: H_2O (50:50)	0,0042	0,48%
Fr-23	Methanol: H_2O (50:50)	0,0041	0,47%
Fr-24	H_2O (100)	0,0076	0,88%

Table S2. References used for BLAST analysis.

Compound	Gene	Situation	Organism	Accession	Reference	Seq
Compactin	mlcH, mlcG, mlcF, mlcA, mlcC, mlcB, mlcD, mlcE, mlcR	Full Cluster	*Penicillium citrinum*	AB072893.1	10.1007/s00438-002-0697-y	NT
Malformin C	mlfA	CDS	*Aspergillus vadensis* CBS 113365	A0A319BQC1.1	10.1038/s41598-018-36561-3	NT
			Aspergillus neoniger CBS 115656	A0A318Z3U0.1	10.1038/s41598-018-36561-3	NT
			Aspergillus niger CBS 513.88	A2QYX4.1	10.1038/s41598-018-36561-3	NT
			Aspergillus niger ATCC 1015	G3XUF0.1	10.1038/s41598-018-36561-3	NT
			Aspergillus luchuensis CBS 106.47	A0A1M3T4K3.1	10.1038/s41598-018-36561-3	NT
			Aspergillus lacticoffeatus CBS 101883	A0A319A6V2.1	10.1038/s41598-018-36561-3	NT
			Aspergillus kawachii IFO 4308	G7XQ31.1	10.1038/s41598-018-36561-3	NT
			Aspergillus homomorphus CBS 101889	A0A395I3F8.1	10.1038/s41598-018-36561-3	NT
Trichothecene	TRI8, TRI7, TRI3, TRI4, TRI6, TRI5, TRI10, TRI9, TRI11, TRI12, TRI13, TRI14,	Full Cluster	*Fusarium sporotrichioides*	AF359360.3	10.1006/fgbi.2001.1256	NT
	TRI8, TRI7, TRI3, TRI4, TRI6, TRI5, TRI10, TRI9, TRI11, TRI12, TRI13, TRI14,		*Gibberella zeae* GZ3639	AF359361.3	10.1006/fgbi.2001.1256	NT
	TRI13	CDS	*Gibberella zeae* HKM136	AY057841.1	10.1006/fgbi.2001.1256	NT
Ochratoxin	ota1-5	Full Cluster	*Aspergillus welwitschiae* ITEM 7468	KX267735	10.3389/fmicb.2016.01412	NT
			Aspergillus welwitschiae ITEM 11945	KX267737	10.3389/fmicb.2016.01412	NT
			Aspergillus niger ITEM 10355	KX267736	10.3389/fmicb.2016.01412	NT
	ota1	CDS	*Aspergillus niger* CBS 513.88	ANI_1_1826134	10.3389/fmicb.2016.01412	AA

	ota2	CDS	*Aspergillus niger* CBS 513.88	An15g07890	10.3389/fmicb.2016.01412	AA
	ota3	CDS	*Aspergillus niger* CBS 513.88	ANI_1_1830134	10.3389/fmicb.2016.01412	AA
	ota4	CDS	*Aspergillus niger* CBS 513.88	ANI_1_1832134	10.3389/fmicb.2016.01412	AA
	ota5	CDS	*Aspergillus niger* CBS 513.88	ANI_1_1836134	10.3389/fmicb.2016.01412	AA
Fumonisin	fum6	CDS	*Aspergillus niger* CBS 513.88	ANI_1_2654014	10.3389/fmicb.2016.01412	AA
	fum10	CDS	*Aspergillus niger* CBS 513.88	ANI_1_2658014	10.3389/fmicb.2016.01412	AA
	fum7	CDS	*Aspergillus niger* CBS 513.88	ANI_1_2660014	10.3389/fmicb.2016.01412	AA
	fum3	CDS	*Aspergillus niger* CBS 513.88	ANI_1_892014	10.3389/fmicb.2016.01412	AA
	fum8	CDS	*Aspergillus niger* CBS 513.88	ANI_1_894014	10.3389/fmicb.2016.01412	AA
	fum13	CDS	*Aspergillus niger* CBS 513.88	ANI_1_2662014	10.3389/fmicb.2016.01412	AA
	fum14	CDS	*Aspergillus niger* CBS 513.88	ANI_1_2664014	10.3389/fmicb.2016.01412	AA
	fum15	CDS	*Aspergillus niger* CBS 513.88	ANI_1_2668014	10.3389/fmicb.2016.01412	AA
	fum1	CDS	*Aspergillus niger* CBS 513.88	ANI_1_2672014	10.3389/fmicb.2016.01412	AA
	fum19	CDS	*Aspergillus niger* CBS 513.88	205909	10.3389/fmicb.2016.01412	AA
	fum21	CDS	*Aspergillus niger* CBS 513.88	225717	10.3389/fmicb.2016.01412	AA
	sdr1	CDS	*Aspergillus niger* CBS 513.88	51907	10.3389/fmicb.2016.01412	AA
	gene B	CDS	*Aspergillus niger* CBS 513.88	35994	10.3389/fmicb.2016.01412	AA
	gene C	CDS	*Aspergillus niger* CBS 513.88	171442	10.3389/fmicb.2016.01412	AA
	gene D	CDS	*Aspergillus niger* CBS 513.88	171846	10.3389/fmicb.2016.01412	AA

Table S3. Assembly metrics, generated with scaffold_stats.pl, for Aspergillus welwitschiae isolates CCMB674 and CBS 139.54.

**Filenames**	**CCMB 674**	**CBS 139.54**
Longest Scaffold	2008406	2162797
Num	244	396
Span	38188994	37511876
Min	1046	1010
Mean	156512	94726
N50	456288	757052
NumN50	23	17
GC	0.483	0.497
**For Runs of Ns (≥10 Ns):**		
Num	9	118
Span	436	33835
N50	96	1190

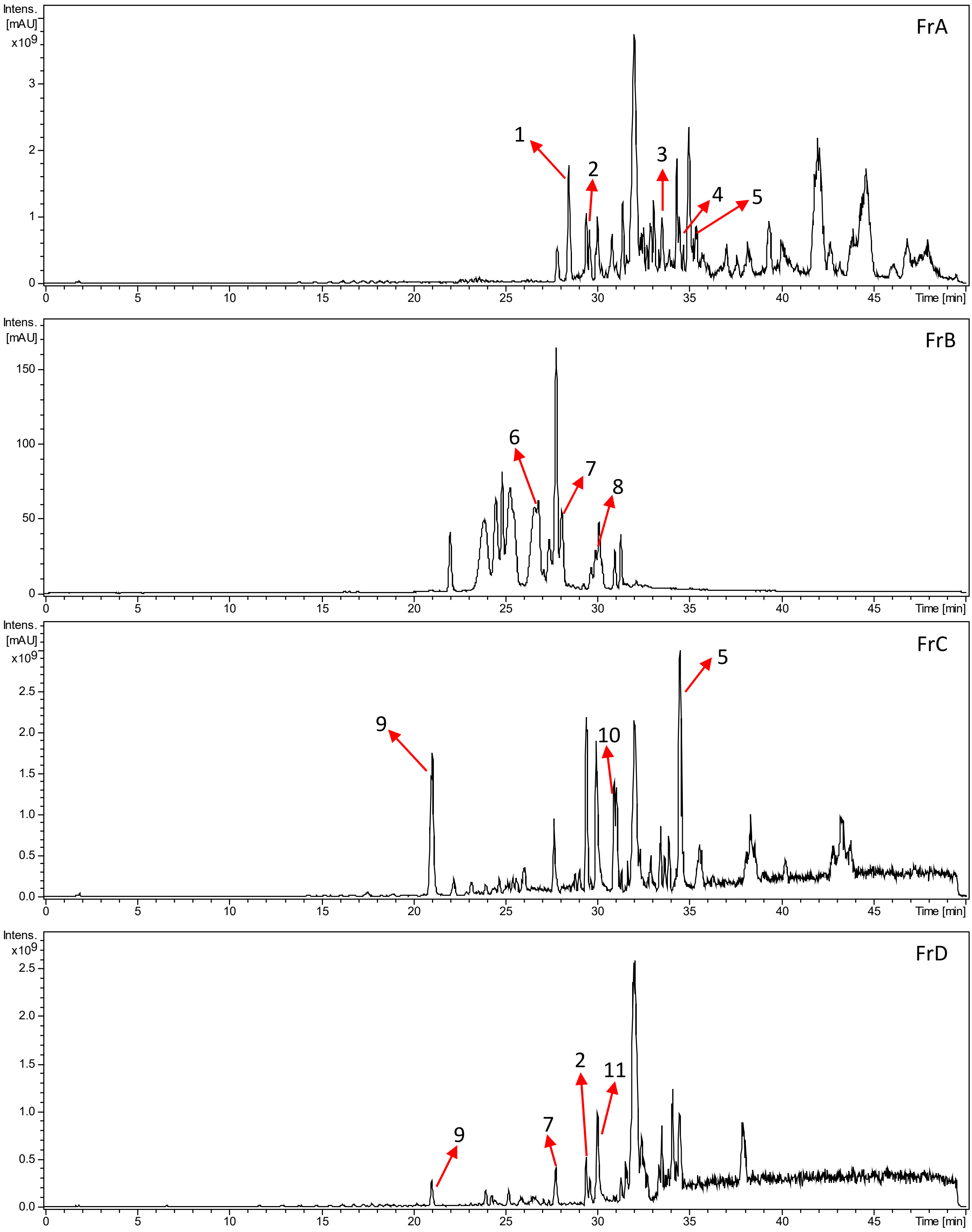

Figure S1. Chromatograms for Fractions A–D.

Mass Spectrometer: Amazon Speed ETD - Bruker

Nebulizer: 27 Psi

HV: 4500V

Dry gas: 12 L/min

ESI+

Temp: 300 ºC

HPLC:

Controler: CBM-20A- Shimadzu

Pump: LC-20AD Shimadzu

Detector: SPD-20A - Shimadzu

Oven: CTO-20A - Shimadzu

Autosampler: SIL 20AC – Shimadzu

Solvent A: H_2O 0,1% Acetic Acid

Solvent B: Methanol

Column: Phenomenex Luna C18 (250x4.6mm – 5um)

Oven Temp: environment

Flux: 1 ml/min

Gradient: 0 min A:B (100:0) 30 min A:B (0:100) 46 min A:B (0:100) 47 min A:B (80:20) 50 min A:B (80:20)

Soma_rede.py

```
# -*- Coding: UTF-8 -*-
#coding: utf-8
import sys
if (len(sys.argv)!=2):
    print ("Please enter file name (Ex: python somaRede.py rede.txt)")
    sys.exit()
nomeArquivo=sys.argv[1]
# nomeArquivo="rede.txt"
arquivoRede=open(nomeArquivo,'r')
resultado=[]
for linha in arquivoRede:
    index=linha.rfind(";")
    nome=linha[0:index]
    achou=0
    for item in resultado:
        if (item[0].find(nome)!=-1):
            item[1]=item[1]+float(linha[index+1:].rstrip())
            achou=1
            break
    if (achou==0):
        aux=[nome,float(linha[index+1:])]
        resultado.append(aux)
arqSaida=open(nomeArquivo[0:nomeArquivo.find(".")]+"_rst.txt",'w')
for item in resultado:
    arqSaida.write(item[0]+";"+str(item[1])+"\n")
    print(item[0]+";"+str(item[1]))
```

```
arqSaida.close()
arquivoRede.close()
```
